# Irreversibility effects in peristaltic transport of hybrid nanomaterial in the presence of heat absorption

**DOI:** 10.1038/s41598-021-98678-2

**Published:** 2021-10-04

**Authors:** Samreen Sheriff, S. Ahmad, N. A. Mir

**Affiliations:** 1 Department of Mathematics and Statistics, Riphah International University, Islamabad, 44000 Pakistan; 2 DBS&H, CEME, National University of Sciences and Technology, Islamabad, 44000 Pakistan

**Keywords:** Biophysics, Materials science, Nanoscience and technology

## Abstract

The nano heat transport has gained much significance in recent era. The micro-level devices are enganged succssfully in diverse fields like electronics, biomedical, navel structures, manufacturing, transportation, and automotive industries in order to improve the heat transfer for cooling and heating. Owing to this fact, the current article illustrates the features of irreversibility and thermal jump in peristaltic transport of hybrid nanoliquid. Here, water is used as base liquid while nanoparticles include polystyrene and graphene oxide. The flow is carried out in a non-uniform channel where the walls of channel flexible nature. Additionally, magnetic field impacts on flow and Joule heating analysis are examined. The aspect featuring heat absorption is introduced. Nanoparticle's shapes effect is also incorporated in flow analysis. Under the consideration of small Rynold number and long wavelength, the relevent equations are reduced by implementing non-dimensional variables. Involved pertinent parameters influence the peristaltic flow characteristics are displayed graphically and discussed concisely. The result indicates that temperature curves are dominant for pure water as compared to P/water nanofluid and P-GO/water hybrid nanofluid. Moreover, the convergent channel shows least entropy effects and extreme effects are noted for divergent case whereas uniform channel stays behind the divergent one.

## Introduction

The enhancement in heat energy leads to a significant contribution in nanotechnology these recent days since it consequences in much savings in the useful energy and cost of treatments of various diseases. The nanoparticles size is up to 100 nm and due to this small structured size, nanoparticles diffusion rate increases which is helpful in diagnose and treatment of tumors. Nanofluids having impactful role in heat transport mechanisms in different configurations lead to more applications that involve the suspension of non-metallic and metallic particles. Nanoparticle is now a prominent domain of investigation for engineers and scientists owing to extensive potential practices in biomedical, electronic and optical fields. Although as drug carriers, satisfactory results have been found by diverse types of nanofluids. Now, recent researchers have started to consider the suspension of different nanoparticles combinations with base fluids and hence this newly formed class of nanofuids is termed as hybrid nanofluids. Such nanofluids are formed due to the combination of two or more particles. This progressive category of nanolfuids has demonstrated great improvement in the thermo-physical properties and stability when compared to single nanofluid. Sheriff et al.^1^ analysed heat transfer with variable fluid features over nanofluid peristaltic flow. Hayat and Nadeem^2^ discussed the enhancement in heat transport process through hybrid nanofluid. Hayat et al.^3^ described the radiative and slip features in hybrid nanofluid flow. Iftikhar et al.^4^ disclosed the nanoparticles shapes and slip features in peristalsis driven motion of magneto hybrid nanoliquid. Zahan et al.^5^ depicted the dissipative and connective impacts in flow of MHD hybrid nanomaterial flow filled in cavity. Awais et al.^6^ explored the hydro-magnetic features in hybrid nanoliquid flow with peristalsis phenomenon. Saleem et al.^7^ discussed the peristaltic movement of hybrid nanoliquid through cilia based tube. Sadaf and Abdelsalam^8^ studied the convective features in hybrid nanoliquid flow through non-unifrom type annulus.

Mechanism of peristaltic motion has increased the interest amongst the investigators recently because of its extensive used in medical engineering and medical field. Phenomenon of peristalsis is natural which emerges due to unprompted expansion and contraction of flexible channel walls. Such mechanism is appeared in swallowing of food, chyme movement, transport of urine, sperm transport, transportation of blood in narrow blood vessel and many others. In bypass operation (coronary), such phenomenon is very helpful for the distribution of blood in heart lung machine. Peristalsis mechanism has proven to be very helpful in transportation of fluid avoiding the fluid from being infacted. Latham^9^ and Shapiro et al.^10^ firstly initiated the work in this direction. In past studies, most of work has been done on peristalsis movement in symmetric channel. Recently Physiologists seen that myometrial contractions phenomenon which describes intra-uterine liquid motion in the human body leads to peristalsis and such phenomenon appears in both uniform and non-uniform channels. The importance to investigate peristaltic motion in non-uniform channel has been exhibited by Akbar et al.^11^. Hayat et al.^12^ discussed the varying viscosity features in hydromagnetic nanomaterial flow with peristalsis phenomenon. Hussain et al.^13^ described peristaltic darcian motion of non-linear radiative fluid under magnetic effects. Prakash et al.^14^ disclosed the peristalsis phenomenon in magneto radiative nanofluid flow. Noreen and Tripathi^15^ depicted the heat transport features in electroosmotic motion through non porous medium under peristaltic pumping impacts. Shukla et al.^16^ described the peristalsis driven motion through inclined non-uniform channel. Abbasi et al.^17^ disclosed the varying liquid features in nanomaterial under the peristalsis mechanism. Imran et al.^18^ explored the peristalsis features in fluid flow via generalized Darcy medium. On the other hand study of MHD phenomenon in peristaltic flow of a fluid attains consiserable attention because of its extensive practical implications in bio engineering and bio-medical sciences, like electrostatic precipitation, cancer therapy, biomechanics, bleeding reduction during surgeries and tumor treatment. Therefore, different studies^6,19–22^ accounted MHD features under various aspects.

In thermo-dynamical system entropy generation is prominent phenomenon which explains the disorderness in a system. In fact it is a significant impact that all real existent processes are irreversible and disorderness in molecules becomes higher with the irreversibility factor of the processes. Thus greater disorder gives rise to larger entropy. Therefore, it is vital to minimize the entropy effect for the improvement in the efficiency of engineering systems. Hence this technique of minimization of entropy generation can be very helpful in order to optimize the engineering systems, including air-conditioning systems, solar collectors, thermal power stations units, heat exchangers and many others. Bejan^23^ is the first who initiated the study upon entropy generation. After that many studies on various entropy generation aspects have been reported by numerous researchers in literature under diverse flow configurations. Hayat et al.^24^ described the the nanoparticles shape and irreversibility analysis in nanomaterial flow with peristalsis. Farooq et al.^25^ discussed the peristalsis features in mixed convective radiative carbon nanotubes flow under the entropy impacts. Abbasi et al.^26^ disclosed the varing viscosity and Hall impacts in peristaltic movement of nanoliquid with entropy generation. Hayat et al.^27^ depicted the endoscopic impacts in peristaltic driven movement through generalized darcian medium under the analysis of entropy phenomenon. Farooq et al.^28^ described the variable features in magnetohydro peristalsis with irreversibility analysis. Zahid et al.^29^ explored the entropy impacts in hybrid nanoliquid under the phenomenon of peristalsis. Hayat et al.^30^ explained the entropy features in peristaltic transport of non-Newtonian liquid with varying thermal conductivity.

Furthermore, the interest toward slip analysis in peristaltic flow with heat transport rate via microscale has enhanced because these phenomena have vast uses in micro-propulsion, chemical separation, micro-thermal technology, cooling process of computer chips and in bio-medical field. It is evident that in various physical situations, no-slip condition can not be sustained; therefore, slip condition necessarily needs to be considered. Slip conditions define when velocity and temperature of fluid near the boundaries is different from an actual velocity and temperature of the boundaries. Some recent researches subject to this phenomenon are presented in Refs.^31–35^.

Motivated by the aforementioned debate, our aim is to examine the peristalsis mechanism in MHD (Magneto-hydrodynamic) hybrid nanoliquid confined in non-uniform channel of flexible wall. Graphene oxide and polystyrene are distributed homogeneously in water to explore the nanofluid features. Heat absorption, Joule heating and thermal jump phenomena are incorporated to scrutinize the heat transportation. Analysis of entropy generation is included and various forms of nanoparticles (sphere, blade, bricks, platelets and cylinders) are discussed. Exact solutions are derived. Results have been presented through graphs corresponding to various emerging parameters.

## Problem formulation

**Figure 1:**
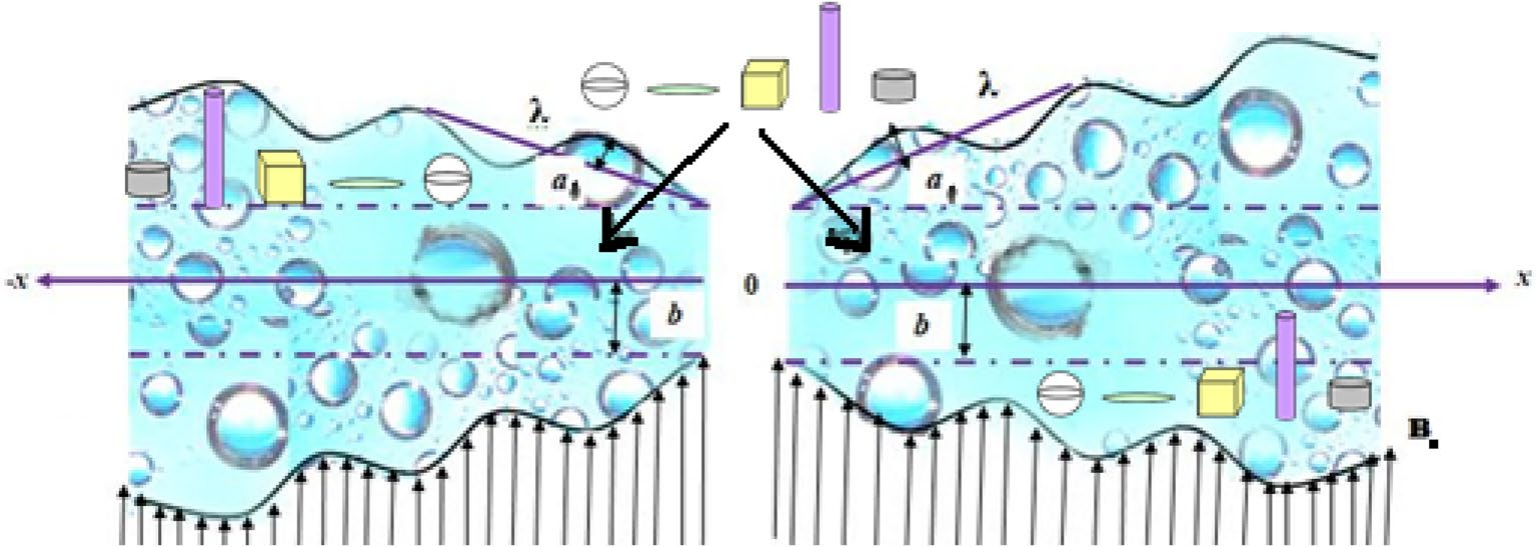
Geometry of the divergent-convergent channel.

Peristalsis driven flow of water based hybrid nanofluid is investigated in a two-dimensional non-uniform flexible channel. The nanoparticles i.e., polystyrene and graphene oxide are considered. Here, Cartesian coordinates $(\overline{x},\overline{y})$ is implemented and thus axes $\overline{x}$ and $\overline{y}$ are taken along the central line and normal position respectively (see Fig. 1). The flow field is influenced by the applied uniform magnetic strength $B_{0}$ while induced magnetic effects are neglected. Irreversibility and nano-shape effects are moreover accounted. Thermal slip, Joule heating and heat absorption phenomena are added in energy equation. The mathematical form of propagating waves along the channel walls is:

1 $$ y = \overline{h} \, \left( {\overline{x},\overline{t}} \right) = a_{1} (x) + b\sin \frac{2\pi }{\lambda }\left( {\overline{x} - c\overline{t}} \right) $$

Using

2 $$ a_{1} (x) = a_{0} + \tilde{m} \, \overline{x} $$

Here $a_{1} (x)$ is defined as the width of the channel taken in half at some axial space $x$ after inlet, $b$ represents the peristaltic wave amplitude, $a_{o}$ gives value of half width at inlet, $\lambda$ stands for wave length, $\overline{t}$ is the time, $c$ denotes the velocity of propagation and $\overline{x}$ specifies the direction of wave progression. The constant $m$ expresses the magnitude of non-uniformity which makes the volume flow rate to vary down the stream length. Indeed, keeping $\tilde{m}$ value equal to zero makes the channel uniform.

Further, fluid flows in labortary frame remains unstable. For stable flow, we switch form labortary frame to wave frame. Thus, transformation is implemented to shift from laboratory to wave frame as:

3 $$ \overline{x} = \overline{X} - c\overline{t},\quad \overline{u} = \overline{U} - c,\quad \overline{p}\left( {\overline{x},\overline{y},\overline{t}} \right) = \overline{P}\left( {\overline{X},\overline{Y},\overline{t}} \right),\quad \overline{y} = \overline{Y},\quad \overline{v} = \overline{V}. $$

The appropriate governing equations for the current problem are as^6,11^:

4 $$ \frac{{\partial \overline{u}}}{{\partial \overline{x}}} + \frac{{\partial \overline{v}}}{{\partial \overline{y}}} = 0,\, $$

5 $$ \rho_{hnf} \left( {\frac{{\partial \overline{u}}}{{\partial \overline{t}}} + \overline{u}\frac{{\partial \overline{u}}}{{\partial \overline{x}}} + \overline{v}\frac{{\partial \overline{u}}}{{\partial \overline{y}}}} \right) = - \frac{{\partial \overline{p}}}{{\partial \overline{x}}} + \frac{\partial }{{\partial \overline{x}}}\left( {2\mu_{hnf} \frac{{\partial \overline{u}}}{{\partial \overline{x}}}} \right) + \frac{\partial }{{\partial \overline{y}}}\left( {\mu_{hnf} \left( {\frac{{\partial \overline{u}}}{{\partial \overline{x}}} + \frac{{\partial \overline{u}}}{{\partial \overline{y}}}} \right)} \right) - \sigma_{hnf} \, {\rm B}_{0}^{2} \overline{u}, $$

6 $$ \rho_{hnf} \left( {\frac{{\partial \overline{v}}}{{\partial \overline{t}}} + \overline{u}\frac{{\partial \overline{v}}}{{\partial \overline{x}}} + \overline{v}\frac{{\partial \overline{v}}}{{\partial \overline{y}}}} \right) = - \frac{{\partial \overline{p}}}{{\partial \overline{x}}} + \frac{\partial }{{\partial \overline{x}}}\left( {2\mu_{hnf} \frac{{\partial \overline{v}}}{{\partial \overline{x}}}} \right) + \frac{\partial }{{\partial \overline{y}}}\left( {\mu_{hnf} \left( {\frac{{\partial \overline{v}}}{{\partial \overline{x}}} + \frac{{\partial \overline{v}}}{{\partial \overline{y}}}} \right)} \right)\,, $$

7 $$ \begin{aligned}  \left( {\rho c_{p} } \right)_{hnf} \left( {\frac{{\partial \overline{T}}}{{\partial \overline{t}}} + \overline{u}\frac{{\partial \overline{T}}}{{\partial \overline{x}}} + \overline{v}\frac{{\partial \overline{T}}}{{\partial \overline{y}}}} \right) &= \kappa_{hnf} \left( {\frac{{\partial^{2} \overline{T}}}{{\partial \overline{y}^{2} }} + \frac{{\partial^{2} \overline{T}}}{{\partial \overline{x}^{2} }}} \right) + \, \mu_{hnf} \left( {2\left( {\left( {\frac{{\partial \overline{u}}}{{\partial \overline{x}}}} \right)^{2} + \left( {\frac{{\partial \overline{v}}}{{\partial \overline{y}}}} \right)^{2} } \right) + \left( {\frac{{\partial \overline{u}}}{{\partial \overline{y}}} + \frac{{\partial \overline{v}}}{{\partial \overline{x}}}} \right)^{2} } \right) \\ &\quad+ \sigma_{hnf} B_{0}^{2} \overline{u}^{2} + \tilde{Q}_{0} , \end{aligned} $$

where $\overline{u},$ $\overline{v}$ depicts velocity components along $\overline{x}$- and $\overline{y}$- directions, $\overline{T}$ represents temperature, pressure is represented by $\overline{p}$, $\tilde{Q}_{0}$ defines heat absorption coefficient, $B_{0}$ specifies magnetic strength, kinematic viscosity is represented by $\upsilon$.

Further, $\rho_{hnf}$, $(\rho c_{p} )_{hnf}$,$\, \sigma_{hnf}$, $\mu_{hnf}$, $\alpha_{hnf}$ and $k_{hnf}$ define effective density, heat capacitance, effective dynamic viscosity, effective electrical conductivity, effective thermal diffusivity and effective thermal conductivity of hybrid nanomaterial are mathematically defined as:

8 $$ \begin{aligned} & T = \frac{{\mu_{hnf} }}{{\mu_{f} }} = \frac{1}{{\left( {1 - \phi_{P} } \right)^{2.5} \left( {1 - \phi_{GO} } \right)^{2.5} }},\quad \rho_{hnf} = \rho_{f} \left( {1 - \phi_{P} } \right)\left( {1 - \phi_{GO} } \right) + \left[ {\phi_{P} \left( {\rho_{sP} /\rho_{f} } \right)} \right] + \phi_{GO} \rho_{sGO} , \\ & \rho_{hnf} \left( {c_{p} } \right)_{hnf} = \left( {1 - \phi_{GO} } \right)\left[ {\left( {1 - \phi_{P} } \right)\rho_{f} \,\left( {c_{p} } \right)_{f} + \phi_{P} \rho_{sP} \left( {c_{p} } \right)_{sP} } \right] + \phi_{GO} \rho_{sGO} \left( {c_{p} } \right)_{sGO} , \\ & \frac{{\sigma_{hnf} }}{{\sigma_{bf} }} = \frac{{\sigma_{sG0} \left( {1 + 2\phi_{G0} } \right) + 2\sigma_{bf} \left( {1 - \phi_{G0} } \right)}}{{\sigma_{sG0} \left( {1 - \phi_{G0} } \right) + \sigma_{bf} \left( {2 + \phi_{G0} } \right)}},\quad \, \sigma_{bf} = \sigma_{f} \left[ {\frac{{\sigma_{sP} \left( {1 + 2\varphi_{P} } \right) + 2\sigma_{f} \left( {1 - \varphi_{P} } \right)}}{{\sigma_{sP} \left( {1 - \varphi_{P} } \right) + \sigma_{f} \left( {2 + \varphi_{P} } \right)}}} \right], \\ & E = \frac{{\sigma_{hnf} }}{{\sigma_{f} }},\quad \alpha_{hnf} = \frac{{k_{hnf} }}{{\left( {\rho c_{p} } \right)_{hnf} }}, \\ & K = \frac{{k_{hnf} }}{{k_{f} }} = \frac{{k_{sGO} + \left( {m_{0} - 1} \right)k_{bf} - \varphi_{GO} \left( {m_{0} - 1} \right)\left( {k_{bf} - k_{sGO} } \right)}}{{k_{sGO} + \left( {m_{0} - 1} \right)k_{bf} + \varphi_{GO} \left( {k_{bf} - k_{sGO} } \right)}} \\ & k_{bf} = k_{f} \left[ {\frac{{k_{sP} + \left( {m - 1} \right)\left( {k_{f} - \varphi_{P} } \right)\left( {m - 1} \right)\left( {k_{f} - k_{sP} } \right)}}{{k_{sP} + \left( {m - 1} \right)k_{f} + \varphi_{P} \left( {k_{f} - k_{sP} } \right)}}} \right], \\ \end{aligned} $$

Here $\varphi_{P} ,\varphi_{GO}$ represent the solid nanoparticle volume fraction for polystyrene and graphene oxide. Thermophysical characteristics of water and nanoparticles as well as shape effects are described in Tables 1 and 2 respectively.

**Table 1:** Thermo-physical features of nanoparticles with base fluid.

Physical characteristics	$c_{p} (J/kgK)$	$\rho (kg/m^{3} )$	$k(W/mK)$	$\sigma (1/K)$
Pure water	1210	1053	0.16	$0.05 \times 10^{ - 5}$
Polystyrene	2430	1115	0.253	$6.7 \times 10^{ - 14}$
Graphene oxide	2090	783	0.145	$1.03 \times 10^{2}$

**Table 2:**
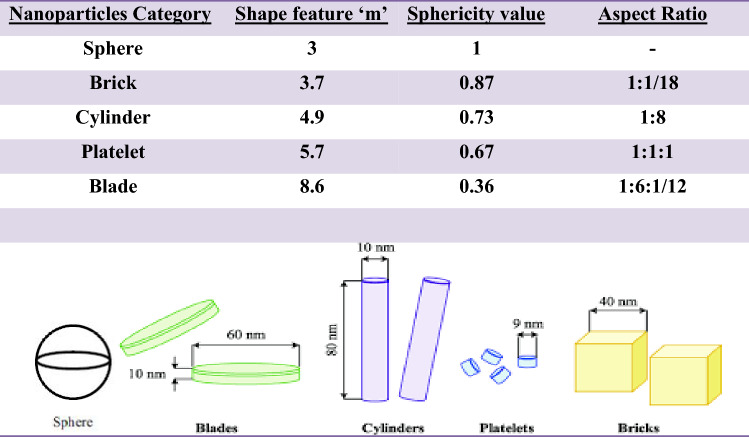
Nanoparticle shape effects.

By introducing the dimensionless variables:

9 $$ \begin{aligned} x & = \frac{{\overline{x}}}{\lambda },\quad u = \frac{{\overline{u}}}{c},\quad y = \frac{{\overline{y}}}{a},\quad v = \frac{{\overline{v}}}{c\delta },\quad p = \frac{{a^{2} \overline{p}}}{{c\lambda \mu_{f} }},\quad \theta = \frac{{\left( {\overline{T} - \overline{T}_{0} } \right)}}{{\overline{T}_{0} }},\quad t = \frac{{c\overline{t}}}{\lambda },\quad \phi = & \frac{b}{a}, \\ \nu & = \frac{\mu }{\rho }{,}\quad {\text{R}}_{{\text{e}}} = \frac{{{\text{ac}}}}{\nu } = \frac{{\rho_{f} (ca)}}{{\mu_{f} }},\quad \delta = \frac{a}{\lambda }{,}\quad \gamma = \frac{{\gamma^{*} }}{a}{,}\quad M^{2} = \frac{{\sigma_{f} B_{0}^{2} a^{2} }}{{\mu_{f} }},\quad m = \frac{{\tilde{m}\lambda }}{a}, \\ {\text{P}}_{{\text{r}}} & = \frac{{\mu c_{p} }}{\kappa } = \frac{{\mu_{f} \left( {c_{p} } \right)_{f} }}{{k_{f} }},\quad {\text{B}}_{{\text{r}}} = {\text{E}}_{{\text{c}}} {\text{ P}}_{{\text{r}}} = \left( {\frac{{a^{2} \mu_{f} }}{{\left( {c_{p} } \right)_{f} \Delta \overline{T}}}} \right){\text{P}}_{{\text{r}}} ,\quad \beta = \frac{{\tilde{Q}_{0} a^{2} }}{{T_{0} k_{f} }}. \\ \end{aligned} $$

and

10 $$ u = \frac{\partial \Psi }{{\partial y}},\quad v = - \delta \frac{\partial \Psi }{{\partial x}},\, $$

we get the follow equations:

11 $$ \frac{\partial p}{{\partial x}} = \frac{\partial }{\partial y}\left( {\left( {\frac{{\mu_{hnf} }}{{\mu_{f} }}} \right)\frac{{\partial^{2} \psi }}{{\partial y^{2} }}} \right) - \, M^{2} \left( {\frac{{\sigma_{hnf} }}{{\sigma_{f} }}} \right) \, \left( {\frac{\partial \psi }{{\partial y}} + 1} \right),\, $$

On differentiating (11), we get equation free of pressure gradient term as:

12 $$ 0 = \, \left( T \right)\frac{{\partial^{4} \psi }}{{\partial y^{4} }} - \left( {M^{2} \left( E \right)} \right)\frac{{\partial^{2} \psi }}{{\partial y^{2} }},\, $$

13 $$ \frac{\partial p}{{\partial y}} = 0,\, $$

14 $$ \left( {\frac{{k_{hnf} }}{{k_{f} }}} \right) \, \frac{{\partial^{2} \theta }}{{\partial y^{2} }} + B_{r} \, \left( T \right) \, \left( {\frac{{\partial^{2} \psi }}{{\partial \overline{y}^{2} }}} \right)^{2} + B_{r} M^{2} \, \left( E \right) \, \left( {\frac{\partial \psi }{{\partial \overline{y}}} + 1} \right)^{2} + \beta = 0,\, $$

with dimensionless conditions at boundary^12^:

15 $$ \left\{ {\begin{array}{*{20}l} {\frac{{\partial^{2} \psi }}{{\partial y^{2} }} = 0,} \hfill & {\quad \psi = 0,\quad at\quad y = 0} \hfill \\ {\frac{\partial \psi }{{\partial y}} = - 1,} \hfill & {\quad \psi = F,\quad at\quad y = h} \hfill \\ \end{array} } \right., $$

16 $$ \left\{ {\begin{array}{*{20}l} {\frac{\partial \theta }{{\partial y}} = 0,} \hfill & {\quad y = 0} \hfill \\ {\theta + \gamma \frac{\partial \theta }{{\partial y}} = 0,} \hfill & {\quad y = h} \hfill \\ \end{array} } \right.. $$

Keeping expression for,

17 $$ y = h = h\left( x \right) = 1 + \tilde{m}x + \phi \sin \left( {2\pi x} \right). $$

Here, $\phi$ represents amplitude ratio, $\gamma$ represents thermal slip parameter, Reynolds number is described by $R_{e}$, $\beta$ defines the heat absorption parameter, $M$ represents Hartmann number, $B_{r}$ represents Brinkman number, $\delta$ represents wave number, $\theta$ denotes dimensionless temperature.

The conditions $\psi (0) = 0$ and $\psi (h) = F$ imposed here have great physical significance as they maintain constant cross-sectional flow rate across the channel walls.

### Solution method

The exact solutions of Eqs. (11)–(14) along with subjected boundary conditions (15) and (16) is formulated as:

18 $$ \psi (x,y) = - \frac{{e^{{A_{2} y}} \left[ {A_{3} \left( { - e^{{A_{2} h}} + e^{{A_{2} (h + 2y)}} } \right) + ye^{{A_{2} y}} \left( {\sqrt T - FA_{1} } \right) - ye^{{A_{2} (2h + y)}} \left( {\sqrt T + FA_{1} } \right)} \right]}}{{\sqrt T + e^{{2hA_{2} }} \left( { - \sqrt T + FA_{1} } \right) + hA_{1} }} $$

19 $$ u(x,y) = - \frac{{e^{{A_{2} y}} \left[ {e^{{A_{2} y}} \left( {\sqrt T - FA_{1} } \right) - e^{{A_{2} (2h + y)}} \left( {\sqrt T + FA_{1} } \right) + A_{4} \left( {e^{{A_{2} h}} + e^{{A_{2} (h + 2y)}} } \right)} \right]}}{{\sqrt T + e^{{2hA_{2} }} \left( { - \sqrt T + FA_{1} } \right) + hA_{1} }} $$

20 $$ \begin{aligned} \theta (x,y) & = \frac{{\left( { - 2B_{r} \left( { - 1 + e^{{4hA_{2} }} } \right) \, \gamma \, A_{5} A_{1}^{3} + \left( {\left( {A_{1}^{4} A_{6} B_{r} } \right)\left( {A_{7} } \right)^{2} + \left( {A_{8} } \right)^{2} TQ_{0} - 2\left( {A_{9} } \right)\sqrt T Q_{0} hA_{1} } \right)\left( {h^{2} - y^{2} + 2h\gamma } \right)} \right)  }}{{\left( {2K\left( {\sqrt T + e^{{2hA_{2} }} \left( { - \sqrt T + FA_{1} } \right) + hA_{1} } \right)^{2} } \right)}} \\ & \quad \frac{{ - A_{{_{1} }}^{2} \left( {B_{r} \left( \begin{gathered} 3 + 8e^{{2hA_{2} }} + 3e^{{4hA_{2} }} - 4e^{{\left( {h - y} \right)A_{2} }} + e^{{2\left( {h - y} \right)A_{2} }} \hfill \\ - 4e^{{\left( {3h - y} \right)A_{2} }} - 4e^{{\left( {h + y} \right)A_{2} }} + e^{{2\left( {h + y} \right)A_{2} }} - 4A_{3}^{2} e^{{\left( {3h + y} \right)A_{2} }} \hfill \\ \end{gathered} \right) - Q_{0} h^{2} \left( {A_{7} } \right)^{2} \left( {h^{2} - y^{2} + 2h\gamma } \right)} \right)}}{{\left( {2K\left( {\sqrt T + e^{{2hA_{2} }} \left( { - \sqrt T + FA_{1} } \right) + hA_{1} } \right)^{2} } \right)}} \\ \end{aligned} $$

where

21 $$ \begin{aligned} T & = \frac{{\mu_{hnf} }}{{\mu_{f} }},\quad E_{c} = \frac{{\sigma_{hnf} }}{{\sigma_{f} }},\quad \alpha_{hnf} = \frac{{k_{hnf} }}{{\left( {\rho c_{p} } \right)_{hnf} }},\quad K = \frac{{k_{hnf} }}{{k_{bf} }}, \\ A_{1} & = M\sqrt {E_{c} } ,\quad A_{2} = \frac{{A_{1} }}{\sqrt T },\quad A_{3} = (F + k)\sqrt T ,\quad A_{4} = (F + h)A_{1} ,\quad A_{5} = \frac{{A\frac{2}{3}}}{\sqrt T }, \\ A_{6} & = (F + k)^{2} ,\quad A_{7} = \left( {1 + e^{{2hA_{2} }} } \right),\quad A_{8} = \left( { - 1 + e^{{2hA_{2} }} } \right),\quad A_{9} = \left( { - 1 + e^{{4hA_{2} }} } \right), \\ \end{aligned} $$

The volumetric rate of flow is assessed by means of:

22 $$ F = \int\limits_{0}^{h\left( x \right)} {\psi \, dy} = \int\limits_{0}^{h\left( x \right)} {\frac{\partial u}{{\partial y}} \, dy} , $$

Axial pressure gradient is expressed by:

23 $$ \frac{dp}{{dx}} = \frac{{(F + h)A_{1}^{3} }}{{ - hA_{1} + \sqrt T \, {\text{Tanh}} [hA_{2} ]}}, $$

We can estimate the value of dimensionless pressure rise by the integral expression:

24 $$ \Delta P = \int\limits_{0}^{1} {\left( {\frac{\partial p}{{\partial x}}} \right)dx} $$

The heat transfer coefficient at the upper wall of the non-uniform channel is defined in the dimensional form:

25 $$ Z = \eta \left| {\frac{\partial \theta }{{\partial y}}} \right|_{y = h} , $$

The mean flow rate which is in relation with flow rate considered in wave frame. This relationship can be presented by:

26 $$ F = Q - 1. $$

## Entropy generation analysis

Heat transfer and viscous impacts of fluid are responsible for the production of thermo-fluidic irreversibility phenomenon. In current investigation entropy generation is influenced by heat transfer phenomenon, viscous dissipation effect and Joule heating. Thus, the dimensional form of the entropy generation is as follows:

27 $$ E_{Gen} = \frac{{\kappa_{hnf} }}{{\overline{T}_{0}^{2} }}\left( {\frac{{\partial^{2} \overline{T}}}{{\partial \overline{y}^{2} }} + \frac{{\partial^{2} \overline{T}}}{{\partial \overline{x}^{2} }}} \right) + \, \frac{{\mu_{hnf} }}{{\overline{T}_{0} }}\left( {2\left( {\left( {\frac{{\partial \overline{u}}}{{\partial \overline{x}}}} \right)^{2} + \left( {\frac{{\partial \overline{v}}}{{\partial \overline{y}}}} \right)^{2} } \right) + \left( {\frac{{\partial \overline{u}}}{{\partial \overline{y}}} + \frac{{\partial \overline{v}}}{{\partial \overline{x}}}} \right)^{2} } \right) + \frac{{\sigma_{hnf} }}{{\overline{T}_{0} }}B_{0}^{2} \overline{u}^{2} ,\,\, $$

The non-dimensional number for entropy generation is expressed as:

$$ E_{T} = \frac{{E_{Gen} }}{{E_{{G_{0} }} }} = \left( {\frac{{k_{hnf} }}{{k_{f} }}} \right) \, \left( {\frac{\partial \theta }{{\partial y}}} \right)^{2} + \frac{{B_{r} \, }}{\Lambda }\left( {\frac{{\mu_{hnf} }}{{\mu_{f} }}} \right) \, \left( {\frac{{\partial^{2} \psi }}{{\partial \overline{y}^{2} }}} \right)^{2} + \frac{{B_{r} M^{2} \, }}{\Lambda }\left( {\frac{{\sigma_{hnf} }}{{\sigma_{f} }}} \right) \, \left( {\frac{\partial \psi }{{\partial \overline{y}}} + 1} \right)^{2} , $$

or

28 $$ E_{T} = E_{H} + E_{F} + E_{J} = E_{H} + E_{B} , $$

Here, $E_{H}$ represents heat transport irreversibility, $E_{B}$ represents fluid friction irreversibility due to combined impacts of Joule heating and viscous dissipation, $B_{r}$ represents Brinkman number, $E_{{G_{0} }}$ defines the characteristics entropy generation rate and $\Lambda$ defines the temperature ration parameters which are given as:

29 $$ E_{{G_{0} }} = \frac{{k_{hnf} }}{{\overline{T}_{0} }}\frac{{\Delta \overline{T}}}{{a^{2} }},\quad \Lambda = \frac{{\Delta \overline{T}}}{{\overline{T}_{0} }},\quad {\text{B}}_{{\text{r}}} = \frac{{a^{2} \mu_{f}^{2} }}{{k_{f} \Delta \overline{T}}}. $$

Furthermore, $B_{e} = E_{H} /E_{T}$ represents Bejan number which relates total irreversibility and irreversibility due to heat transfer and mathematically expressed as:

$$ B_{e} = \frac{{E_{H} }}{{E_{T} }} = \frac{{\left( {\frac{{k_{hnf} }}{{k_{f} }}} \right) \, \left( {\frac{\partial \theta }{{\partial y}}} \right)^{2} }}{{\left( {\frac{{k_{hnf} }}{{k_{f} }}} \right) \, \left( {\frac{\partial \theta }{{\partial y}}} \right)^{2} + \frac{{B_{r} \, }}{\Lambda }\left( {\frac{{\mu_{hnf} }}{{\mu_{f} }}} \right) \, \left( {\frac{{\partial^{2} \psi }}{{\partial \overline{y}^{2} }}} \right)^{2} + \frac{{B_{r} M^{2} \, }}{\Lambda }\left( {\frac{{\sigma_{hnf} }}{{\sigma_{f} }}} \right) \, \left( {\frac{\partial \psi }{{\partial \overline{y}}} + 1} \right)^{2} }}. $$

## Discussion

This section predicts the influence of pertinent parameters on temperature field, entropy generation, axial flow velocity, pressure gradient and pressure rise for different values of the nanoparticle forms (sphere, blade, bricks, platelets and cylinders). Hence figures have been plotted. In these figures, pure water ($\varphi_{P} = 0 = \varphi_{GO}$) is represented by solid lines. The concentration of polystyrene $\varphi_{P}$ is taken fixed $\left( {\phi_{P} = 0.1} \right)$ with varying concentration of graphene oxide ranges $0 \le \varphi_{GO} \le 0.1$ to form the P-GO/water hybrid nanofluid. Moreover, the case for homogeneously concentrated polystyrene and graphene oxide nanoparticles is also discussed.

### Characteristics of thermal distribution

**Figure 2:**
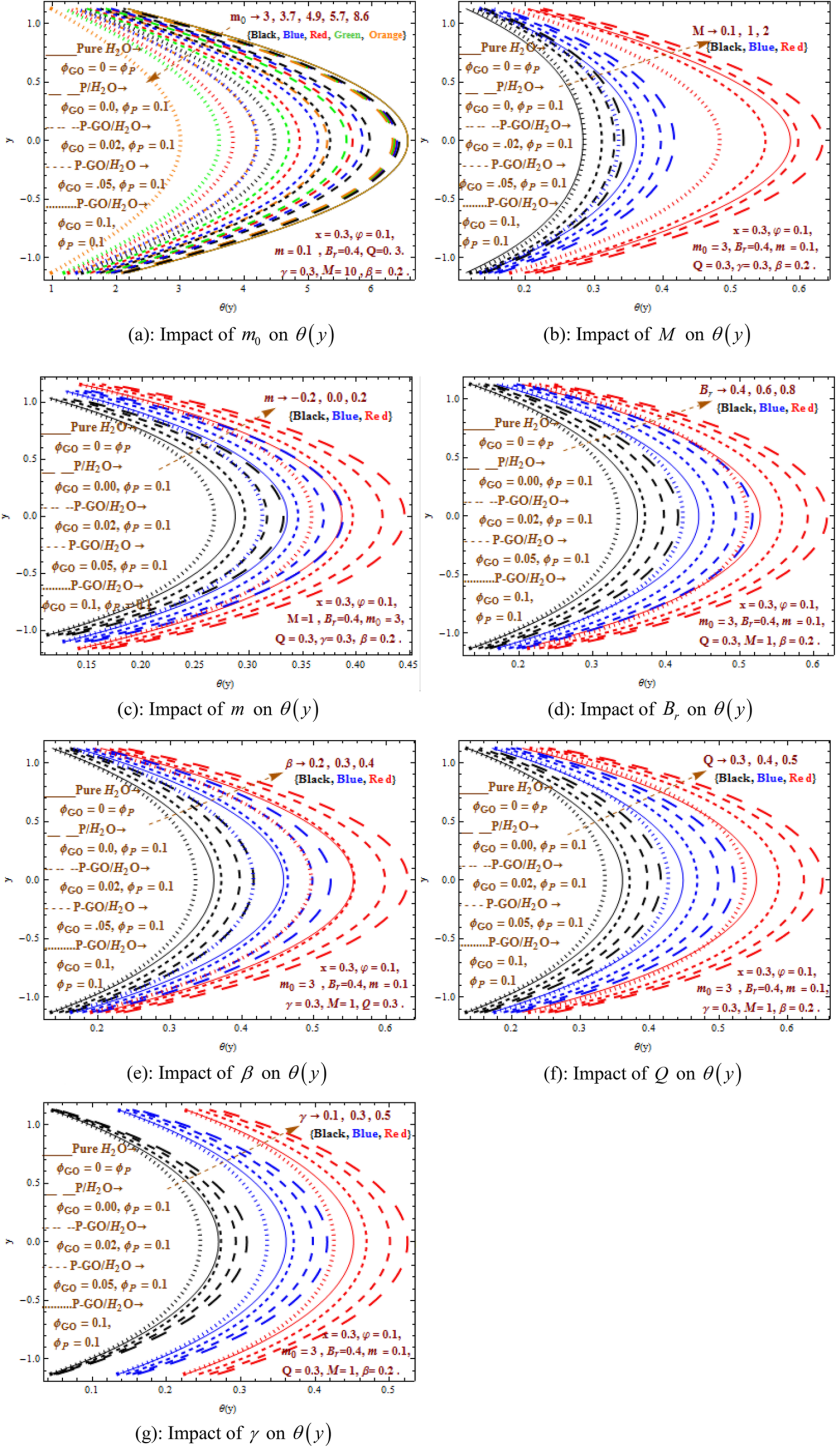
(**a**) Impact of $m_{0}$ on $\theta \left( y \right)$. (**b**) Impact of $M$ on $\theta \left( y \right)$. (**c**) Impact of $m$ on $\theta \left( y \right)$. (**d**) Impact of $B_{r}$ on $\theta \left( y \right)$. (**e**) Impact of $\beta$ on $\theta \left( y \right)$. (**f**) Impact of $Q$ on $\theta \left( y \right)$. (**g**) Impact of $\gamma$ on $\theta \left( y \right)$.

Figure 2a–g illustrates the outcomes of $M$(Hartman number), $m$ (non-uniformity parameter), $\beta$ (heat absorption parameter), $Q$ (flow rate), $B_{r}$ (Brinkman number),$m_{0}$(shape effecting parameter), and $\gamma$ (thermal slip parameter) on temperature field under the nanoparticles shape effect analysis. The nanoparticles shape analysis (sphere, bricks, cylinder, platelet and blade) predicts that temperature curve declines for the different values of $m_{0}$. The temperature curves are dominant for pure water as compared to P/water nanofluid and P-GO/water hybrid nanofluid in the ranges $0 \le 0.02 \le 0.05 \le \varphi_{GO} \le 0.1$. While the least heat transfer is observed for homogeneously distributed nanoparticles i.e. $\varphi_{P} = 0.1 = \varphi_{GO}$. Furthermore, it is noticed that temperature field is increasing function of the parameters $M,m,\beta ,Q,B_{r} ,\gamma$. Here, it is perceived that temperature rise shows less dominancy for P/water nanofluid as compared to P-GO/water hybrid nanofluid when $\phi_{GO}$ ranges $0 \le 0.02 \le 0.05 \le \varphi_{GO} \le 0.1$. Moreover, results found that P-GO/water hybrid nanofluid acts as best coolant for almost all cases when the both nanoparticles are homogeneously distributed into base fluid i.e. $\varphi_{P} = 0.1 = \varphi_{GO}$. Thus, it is of great importance in mechanism of mechanical equipment where coolants are used. The shape effect phenomenon is illustrated for different shapes and it is reflected that changing values of $\left( {m_{0} = 3,3.7,4.9,5.7,8.6} \right)$ (see Table 2) give impact of different nanoparticles shapes on temperature field. Here, it is witnessed that the sphere shape nanoparticle produces more heat rather than the rest of the nanoparticals shapes. Brick shape nanoparticles are more dominant as compared to cylinder and platelet shape nanoparticles whereas blade shape nanoparticle gives minimum temperature. A throughout upsurge in temperature field is seen when $M,m,B_{r} ,\beta ,Q,\gamma$ are increased. Larger Hartman number intensifies the Lorentz force which produces more resistance to the fluid and consequently more heat is generated. Hence, temperature grows. Dominant behavior is revealed in temperature field in channel with slop value $m > 0$ in comparison to the uniformity channel $\left( {m = 0} \right)$ whereas minimum temperature is achieved in the channel with slop value $m < 0$. In fact, convergent channel generates less heating as compared to uniform and divergent channel. Brinkman number is the outcome of Prandtl number and Eckert number where one describes relationship among momentum and thermal diffusivities and the other defines resistance production between fluid particles. Physically, Brinkman number is demonstrated as the quantity to measure irreversibility of the fluid resistance. In fact, enlarge Brinkman number gives greater drag force among fluid particle and as the consequence more heat is generated. Thus, temperature field grows. A significant heat generation is seen on growing values of heat parameter, flow rate and thermal slip $\beta ,Q,\gamma$. It should be described here that the current results are acquired as in^36^ by an exact solutions. For the purpose of comparison, good agreement can be noticed between our exact results displayed in Fig. 2e and those which are obtained in Fig. 2a with $m = 0$, $Br = 0$, $\phi_{Go} = 0$ by past study^36^. Owing the fact, the comparision may be indicated that our outcomes, and exact solutions acquired in^33^ are in excellent agreement.

### Characteristics of entropy generation

**Figure 3:**
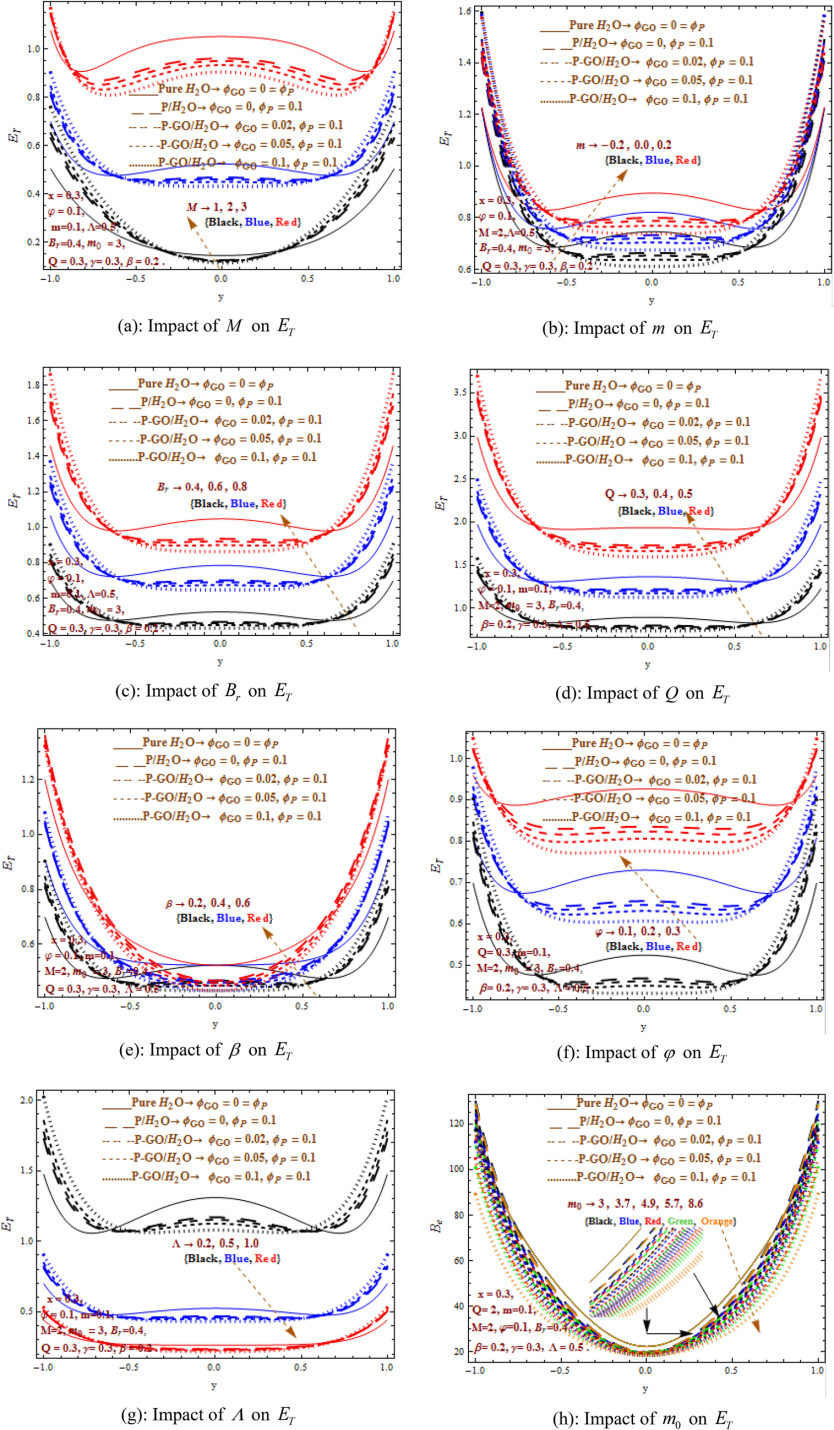
(**a**) Impact of $M$ on $E_{T}$. (**b**) Impact of $m$ on $E_{T}$. (**c**) Impact of $B_{r}$ on $E_{T}$. (**d**) Impact of $Q$ on $E_{T}$. (**e**) Impact of $\beta$ on $E_{T}$. (**f**) Impact of $\varphi$ on $E_{T}$. (**g**) Impact of $\Lambda$ on $E_{T}$. (**h**) Impact of $m_{0}$ on $E_{T}$.

Figure 3a–h states the characteristics of physical parameters i.e. Hartman number $(M)$, non-uniformity parameter $(m)$, heat absorption parameter $(\beta )$, temperature ration parameter $\Lambda$, flow rate $Q$, Brinkman number $B_{r}$, shape effecting parameter $m_{0}$, amplitude ratio $\varphi$ on entropy generation $\left( {E_{T} } \right)$ considering different shapes (sphere, bricks, cylinder, platelet and blade). The results show least value of entropy generation at the central protion of channel while entropy generation elevates near the walls under the shape effects. In entropy generation case pure water dominates the P/water nanofluid and P-G0/water hybrid nanofluid whereas P-G0/water hybrid nanofluid attains lowest position throughout the entropy generation analysis. With the varying values of $0 \le 0.02 \le 0.05 \le \varphi_{GO} \le 0.1$, hybrid nanofluid is more dominant in comparison to the homogeneously distributed nanoparticles case i.e. $\varphi_{P} = 0.1 = \varphi_{GO}$. It is also observed that entropy generation $\left( {E_{T} } \right)$ shows diverse trend throughout central and near the wall regions when $M,m,B_{r} ,\Lambda ,Q,\beta ,\varphi$ are increased with different values of $0 \le 0.02 \le 0.05 \le \varphi_{GO} \le 0.1$. Moreover, intensification in $M,m,B_{r} Q,\beta ,\varphi$ results in increasing entropy generation in both regions while entropy generation produces a decrement when $\Lambda ,m_{0}$ is increased. The shape parameter $\left( {m_{0} = 3,3.7,4.9,5.7,8.6} \right)$ defines the different shapes of nanoparticles and its effects on entropy generation. The spherical shape nanoparticle shows maximum entropy generation while blade shape nanoparticle displays minimum magnitdue of entropy generation due to maximum heat transfer. The spherical shape nanoparticle shows maximum entropy generation while blade shape nanoparticle displays minimum magnitude of entropy generation due to maximum heat transfer. The cylinder shape nanoparticle exists between brick and platelet nanoparticles. The entropy generation $\left( {E_{T} } \right)$ curves are affected intensely by large change in parameters $M,B_{r} Q$ which result in upturn of results where maximum value is attained at the wall and least at the center. The convergent channel shows least entropy effects and extreme effects is noted for divergent case whereas uniform channel stays behind the divergent one.

### Characteristics of Bejan number

**Figure 4:**
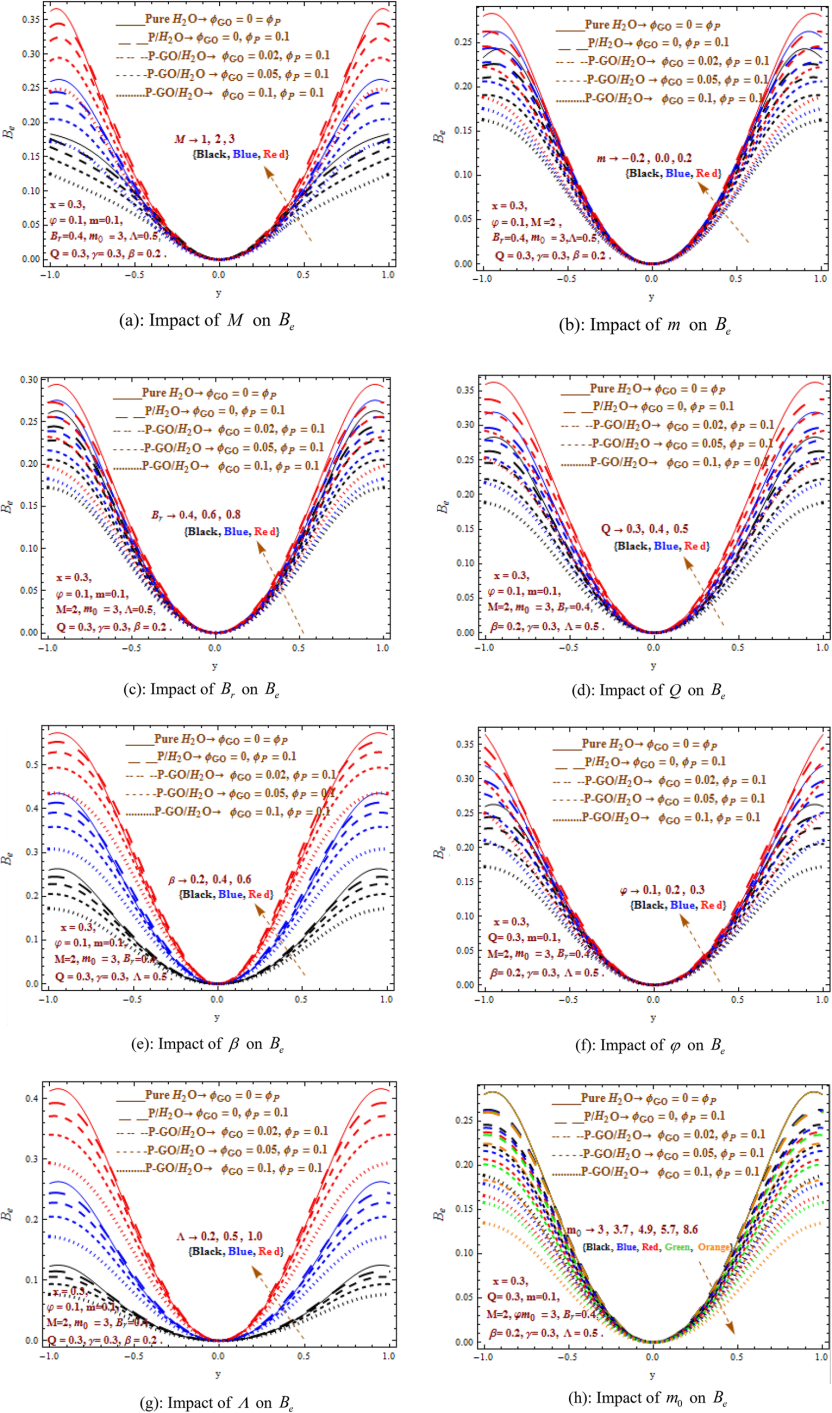
(**a**) Impact of $M$ on $B_{e}$. (**b**) Impact of $m$ on $B_{e}$. (**c**) Impact of $B_{r}$ on $B_{e}$. (**d**) Impact of $Q$ on $B_{e}$. (**e**) Impact of $\beta$ on $B_{e}$. (**f**) Impact of $\varphi$ on $B_{e}$. (**g**) Impact of $\Lambda$ on $B_{e}$. (**h**) Impact of $m_{0}$ on $B_{e}$.

Figure 4a–h reflects the graphical investigations of energing parameters i.e. Hartman number (M), non-uniformity parameter (m), heat absorption parameter (β), temperature ration parameter $\Lambda$, flow rate $Q$, Brinkman number $B_{r}$, shape effecting parameter $m_{0}$, amplitude ratio $\varphi$ on Bejan number $\left( {B_{e} } \right)$. Here, with different values of $m_{0}$, similar trend is observed in Bejan number $\left( {B_{e} } \right)$ as for entropy generation $\left( {E_{T} } \right)$. With increased values of $M,m,B_{r} ,\Lambda ,Q,\beta ,\varphi$, Bejan number $\left( {B_{e} } \right)$ attains more elevation in case of pure water as compared to P/water nanofluid and P-GO/water hybrid nanofluid, where the allowable used ranges for graphene oxide particle is $0 \le 0.02 \le 0.05 \le \varphi_{GO} \le 0.1$. Moreover, we found an increase in Bejan number $\left( {B_{e} } \right)$ subjected to growing parameters $M,m,\beta ,\Lambda ,Q,B_{r} ,\varphi$ whereas the parameter $m_{0}$ decrements the Bejan number $\left( {B_{e} } \right)$. Moreover, it concludes that entire entropy generated leads over the entropy generated as a consequence of heat transference irreversibility.

### Characteristics of velocity

**Figure 5:**
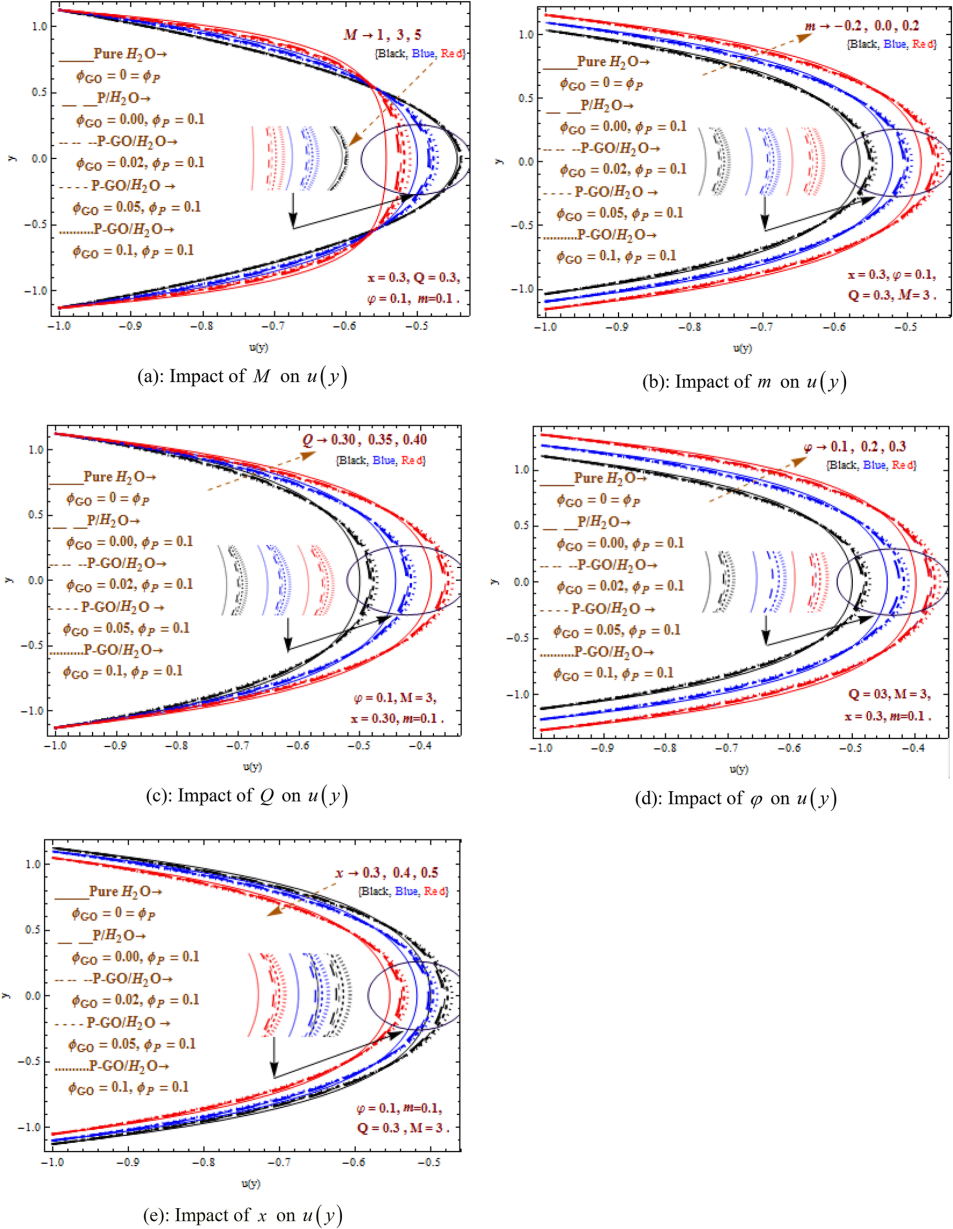
(**a**) Impact of $M$ on $u\left( y \right)$. (**b**) Impact of $m$ on $u\left( y \right)$. (**c**) Impact of $Q$ on $u\left( y \right)$. (**d**) Impact of $\varphi$ on $u\left( y \right)$. (**e**) Impact of $x$ on $u\left( y \right)$.

Figure 5a–e presents axial velocity field against pertinent physical parameters $M$ (Hartman number), $m$ (non-uniformity parameter), $Q$ (flow rate), $\varphi$ (amplitude ratio), $x$ (axial inlet). A diverse trend of the velocity profile in the central region and near the walls is witnessed. Here, in the central area, minimum velocity is achieved for pure water and maximum velocity is found in case of homogeneously distributed P-GO/water $\left( {\varphi_{P} = 0.1 = \varphi_{GO} } \right)$ hybrid nanofluid. Moreover it is apparent that a rise in velocity occurs for P-GO/water hybrid nanofluid in comparison to P/water nanofluid in the central region with graphene oxide ranges $0 \le 0.02 \le 0.05 \le \varphi_{GO} \le 0.1$. However, opposite trend is observed near the walls. Larger $m,Q,\phi$ intensifies the axial flow velocity in the central region while decrement is noted in velocity near the walls. On the contrary reverse behavior is displayed for rising parameters $M,x$. Furthermore, decrement is noticed in velocity for larger $M$ while growing $M$ shows increasing trend of velocity in the vinicity of walls. Physically, it explains the resistance to velocity due to increased Lorentz forces. The non-uniformity parameter $m$ describes the convergent ($m < 0$) and divergent ($m > 0$) flows channel and $m = 0$ respresents straight flow channel when no change. The flow velocity in divergent channel shows minimum value while leaving the divergent channel, velocity attains highest magnitude.

### Characteristics of pressure distribution

**Figure 6:**
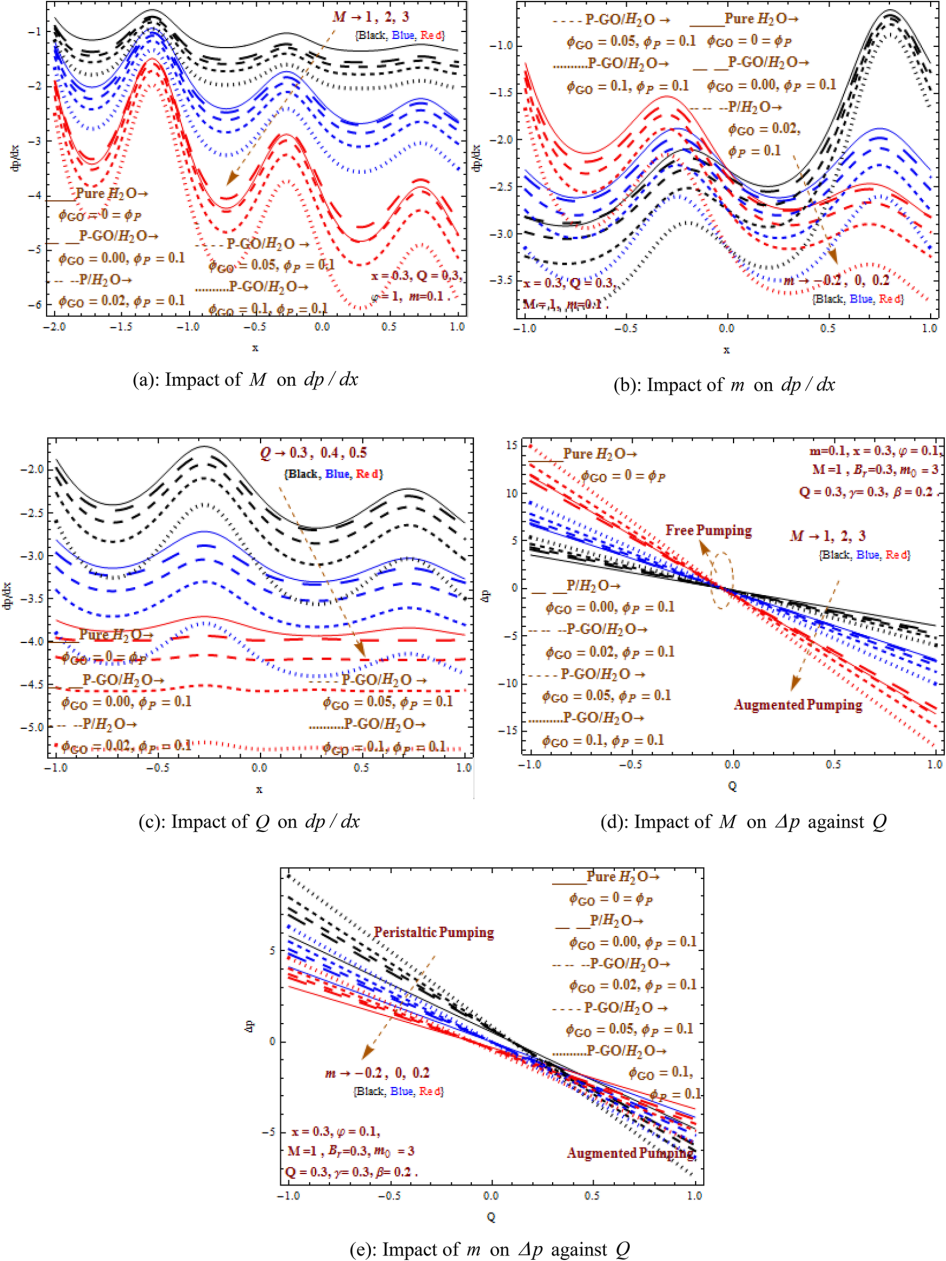
(**a**) Impact of $M$ on $dp/dx$. (**b**) Impact of $m$ on $dp/dx$. (**c**) Impact of $Q$ on $dp/dx$. (**d**) Impact of $M$ on $\Delta p$ against $Q$. (**e**) Impact of $m$ on $\Delta p$ against $Q$.

The pressure gradient $dp/dx$ curves along wave length are observed in Fig. 6a–c with increased values of $M,m,Q$. The pressure gradient shows greatest magnitude for pure water while it gives low magnitude for homogeneously distributed P-GO/water $\left( {\varphi_{P} = 0.1 = \varphi_{GO} } \right)$ hybrid nanofluid. It is also noticed that P/water nanofluid attains higher magnitude when compared with P-GO/water hybrid nanofluid. On the other hand, pressure greadient is decreasing function of $M,m,Q$. Sustainability of greater pressure gradient has implication in medical applications due to its impact on the effectiveness of medicinal delivery. Thus, P/water nanofluid stands at better performance level with higher magnitude in comparison to P-GO/water hybrid nanofluid.

Figure 6d–e depicts the flow parameter influences on pressure rise per wavelength. It is revealed that increasing flow rate decreases the pressure constantly. The pressure rise distribution comprises of three regions namely peristaltic pumping region $\left( {\Delta p > 0} \right)$, free pumping region $\left( {\Delta p = 0} \right)$ and retrograde (augmented) pumping $\left( {\Delta p < 0} \right)$. It is noticed that pressure rise is directly proportional to enhanced magnetic field in the pumping region while it inversely relates in the augmented region. Also it can be observed that P-GO/water hybrid nanofluid achieves the highest magnitude of pressure rise in the pumping region followed by P/water nanofluid and pure water stays at the last with least magnitude of pressure rise. Moreover, higher Hartman number $M$ illustrates opposite variations in pressure rise in comparison to large non-uniformity parameter $m.$ Furthermore, it can be noted that increasing Hartman number enhances the presure rise in the pumping region. Due to the presence of Lorentz forces resistance in movement is produced and as a consequence gives rise in pressure rise. Pressure rise behaves reversely in pumping and augmented regions when non-uniformity parameter is increased. This can be easily seen in the pressure rise graph showing pressure rise magnitude to be lesser for the convergent channel and greater magnitude for the divergent channel due to greater flows.

### Characteristics of trapping

**Figure 7:**
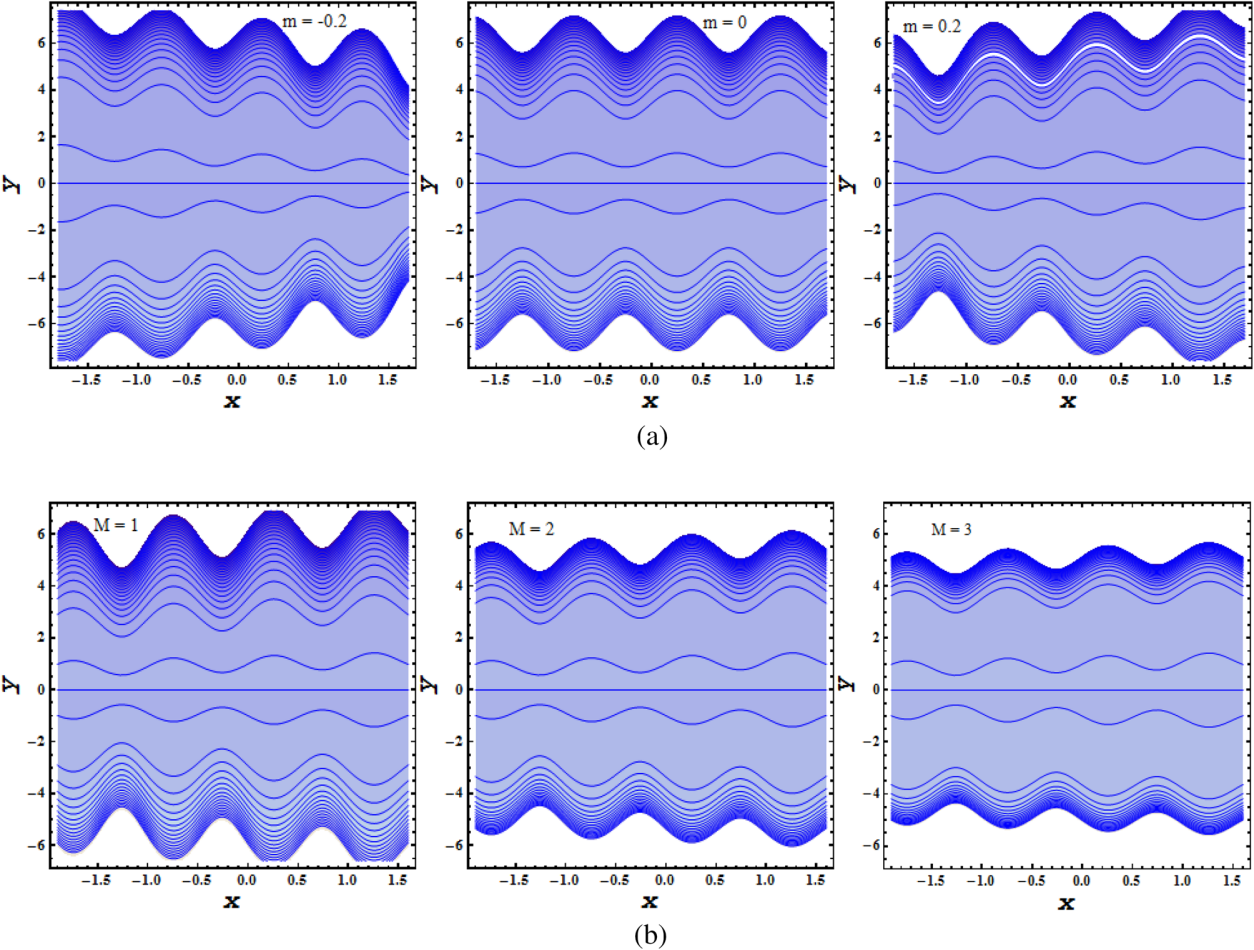
(**a**) Impact of non-unifromity parameter $m$ on the Stream function $\psi$. (**b**) Impact of Hartmann number $M$ on the Stream function $\psi$.

The trapping $\psi$ phenomenon is observed in Fig. 7a,b with increased values of $m, \, M$. Figure 7a portrays the variation affect of non uniformity parameter reflecting convergent channel, uniform channel and divergent channel where flow pattern is concentrated near the wall area and less dense in the central region. The impact of hydromagnetic characteristics due to presence of magnetic field gives rise to Lorentz force bringing retardation effect to flow size which can be seen in the Fig. 7b.

Moreover, Tables 3, 4, 5 and 6 are constructed to see the effects of different shape properties, their heat transfer impacts at the wall and non-uniformity effects on the shape variation regarding P/water nanofluid, P-GO/water hybrid nanofluid and base fluid. Table 3 depicts that different nanoparticle shapes decreases the temperature field for base fluid and all others nanpofluids.Table 4 illustrates that the velocity field corresponds to base and nanofluids increases form divergent to convergent region. Tables 5 and 6 explore the heat transfer rate in both convergent and divergent channel under the analysis of nanoparticles shape effects. Heat transfer shows diverse trend for different nanoparticles shapes and all nanofluids.

**Table 3:** Temperature field analysis for shape effect for P/water nanofluid, P-GO/water hybrid nanofluid and P-GO/water hybrid nanofluid keeping $M = 10,m = 0.1,Q = 0.3,\beta = 0.2,$ $\gamma = 0.3,\varphi = 0.1,B_{r} = 0.4$ for divergent channel.

X	Shape m_0_	0.0	0.3	0.6	0.9	1.125
**Water**						
$$\begin{gathered} \varphi_{P} = 0 \hfill \\ \varphi_{GO} = 0 \hfill \\ \end{gathered}$$	m0 = 3, 3.7, 4.9, 5.7, 8.6	6.56023	6.24171	5.28675	3.70739	2.18297
**P-nanofluid**						
$$\begin{gathered} \varphi_{P} = 0.1 \hfill \\ \varphi_{GO} = 0.0 \hfill \\ \end{gathered}$$	m_0_ = 3	6.42579	6.10915	5.16125	3.60606	2.1245
	m_0_ = 3.7	6.3915	6.07655	5.13371	3.58682	2.11317
	m_0_ = 4.9	6.35943	6.04606	5.10795	3.56882	2.10256
	m_0_ = 5.7	6.34663	6.03389	5.09767	3.56164	2.09833
	m_0_ = 8.6	6.32202	6.01049	5.0779	3.54783	2.09019
**P-G0 hybrid**						
$$\begin{gathered} \varphi_{P} = 0.1 \hfill \\ \varphi_{GO} = 0.05 \hfill \\ \end{gathered}$$	m_0_ = 3	5.3463	5.08079	4.28694	2.99075	1.7628
	m_0_ = 3.7	5.1539	4.89795	4.13267	2.88313	1.69936
	m_0_ = 4.9	4.8708	4.62891	3.90566	2.72476	1.60602
	m_0_ = 5.7	4.70873	4.47013	3.77169	2.6313	1.55093
	m_0_ = 8.6	4.19385	3.99558	3.36285	2.34607	1.38281
**P-G0 hybrid**						
$$\begin{gathered} \varphi_{P} = 0.1 \hfill \\ \varphi_{GO} = 0.1 \hfill \\ \end{gathered}$$	m_0_ = 3	4.47643	4.25211	3.58262	2.49593	1.47206
	m_0_ = 3.7	4.2073	3.99647	3.36723	2.34587	1.38356
	m_0_ = 4.9	3.8251	3.63343	3.06135	2.13277	1.25788
	m_0_ = 5.7	3.6098	3.42898	2.8909	2.01276	1.1871
	m_0_ = 8.6	3.00412	2.85395	2.40429	1.67501	0.9879

**Table 4:** Velocity field variation for convergent/uniform/divergent channel effect analysis on P/water nanofluid, P-GO/water hybrid nanofluid keeping $M = 3,$ $Q = 0.3,\varphi = 0.1$ values fixed.

Slope effect m	− 0.2	− 0.1	0.0	0.1	0.2
**Pure water**					
$$\begin{gathered} \varphi_{P} = 0.0 \hfill \\ \varphi_{GO} = 0.0 \hfill \\ \end{gathered}$$					
X = 0.0	− 0.566022	− 0.542562	− 0.520692	− 0.500277	− 0.481195
X = 0.3	− 0.584484	− 0.560204	− 0.537463	− 0.516151	− 0.496165
X = 0.6	− 0.65586	-0.628491	− 0.602303	− 0.577522	− 0.554043
X = 1.095 + 0.3 m	$\left. { - 1} \right|_{X = 1.03511}$	$\left. { - 1} \right|_{X = 1.06511}$	$\left. { - 1} \right|_{X = 1.09511}$	$\left. { - 1} \right|_{X = 1.12511}$	$\left. { - 1} \right|_{X = 1.15511}$
**P-nanofluid**					
$$\begin{gathered} \varphi_{P} = 0.1 \hfill \\ \varphi_{GO} = 0.0 \hfill \\ \end{gathered}$$					
X = 0.0	− 0.552875	− 0.52833	− 0.50541	− 0.483979	− 0.463918
X = 0.3	− 0.576596	− 0.551307	− 0.527553	− 0.505230	− 0.484242
X = 0.6	− 0.660987	− 0.633049	− 0.606330	− 0.588330	− 0.556547
X = 1.095 + 0.3 m	$\left. { - 1} \right|_{X = 1.03511}$	$\left. { - 1} \right|_{X = 1.06511}$	$\left. { - 1} \right|_{X = 1.09511}$	$\left. { - 1} \right|_{X = 1.12511}$	$\left. { - 1} \right|_{X = 1.15511}$
**P-G0 hybrid**					
$$\begin{gathered} \varphi_{P} = 0.1 \hfill \\ \varphi_{GO} = 0.05 \hfill \\ \end{gathered}$$					
X = 0.0	− 0.547894	− 0.522899	− 0.499534	− 0.477668	− 0.457181
X = 0.3	− 0.573696	− 0.548009	− 0.52385	− 0.501117	− 0.479716
X = 0.6	− 0.660987	− 0.634934	− 0.608022	− 0.582289	− 0.557728
X = 1.095 + 0.3 m	$\left. { - 1} \right|_{X = 1.03511}$	$\left. { - 1} \right|_{X = 1.06511}$	$\left. { - 1} \right|_{X = 1.09511}$	$\left. { - 1} \right|_{X = 1.12511}$	$\left. { - 1} \right|_{X = 1.15511}$
**P-G0 hybrid**					
$$\begin{gathered} \varphi_{P} = 0.1 \hfill \\ \varphi_{GO} = 0.1 \hfill \\ \end{gathered}$$					
X = 0.0	− 0.543201	− 0.517764	− 0.493696	− 0.471657	− 0.450739
X = 0.3	− 0.571005	− 0.544936	− 0.520385	− 0.497252	− 0.475446
X = 0.6	− 0.664967	− 0.636765	− 0.609689	− 0.583750	− 0.558946
X = 1.095 + 0.3 m	$\left. { - 1} \right|_{X = 1.03511}$	$\left. { - 1} \right|_{X = 1.06511}$	$\left. { - 1} \right|_{X = 1.09511}$	$\left. { - 1} \right|_{X = 1.12511}$	$\left. { - 1} \right|_{X = 1.15511}$

**Table 5:** Heat transfer rate $$z = \eta \theta_{y}$$ at wall for P-nano fluid and P-GO hybrid nanofluid varying (non-uniformity) convergent/uniform/divergent channel over shape effect fixing values $$M = 1,x = 0.3,Q = 0.3,\beta = 0.2,\gamma = 0.3,\phi = 0.1,B_{r} = 0.4,$$$$\varphi_{P} = 0.1.$$

$\varphi_{GO}$	$m$	$h_{x} \theta_{y} (x,h)$ m_0_ = 3 $(Sphere)$	$h_{x} \theta_{y} (x,h)$ m_0_ = 3.7 $(Brick)$	$h_{x} \theta_{y} (x,h)$ m_0_ = 4.9 $(Cylinder)$	$h_{x} \theta_{y} (x,h)$ m_0_ = 5.7 $(Platelet)$	$h_{x} \theta_{y} (x,h)$ m_0_ = 8.6 $(Blade)$
0.00	− 0.1	0.1569780	0.1561410	0.155357	0.1550450	0.1544430
	0.0	0.1043780	0.1038210	0.103300	0.1030920	0.1026920
	0.1	0.0508753	0.0506038	0.0503499	0.0502486	0.0500537
0.05	− 0.1	0.1425840	0.1374530	0.1299002	0.1254470	0.1118480
	0.0	0.0946780	0.0912709	0.0862574	0.0832987	0.0742693
	0.1	0.0460722	0.0460722	0.0419945	0.0405348	0.0361409
0.10	− 0.1	0.1313060	0.1234120	0.1122010	0.1058880	0.0881193
	0.0	0.0870713	0.0818365	0.0744023	0.0702159	0.0584334
	0.1	0.0423006	0.0397574	0.0361458	0.0341120	0.0283899

**Table 6:** Heat transfer rate $$z = \eta \theta_{y}$$ at wall h for water, P/water nano fluid , P-G0/water hybrid nano fluid varying non-uniformity parameter fixing $$m_{0} = 3(sphere),$$$$x = 0.3,\gamma = 0.1.$$

$m$	$M$	$B_{r}$	$\phi$	$\beta$	$Q$	$h_{x} \theta_{y} (x,h)$ $$\left( \begin{gathered} \varphi_{P} = 0, \hfill \\ \varphi_{GO} = 0 \hfill \\ \end{gathered} \right)$$	$h_{x} \theta_{y} (x,h)$ $$\left( \begin{gathered} \varphi_{P} = 0.1, \hfill \\ \varphi_{GO} = 0 \hfill \\ \end{gathered} \right)$$	$h_{x} \theta_{y} (x,h)$ $$\left( \begin{gathered} \varphi_{P} = 0.1, \hfill \\ \varphi_{GO} = 0.05 \hfill \\ \end{gathered} \right)$$	$h_{x} \theta_{y} (x,h)$ $$\left( \begin{gathered} \varphi_{P} = 0.1, \hfill \\ \varphi_{GO} = 0.1 \hfill \\ \end{gathered} \right)$$
− 0.1	**1**	**0.4**	**0.1**	0.2	0.3	0.1309090	0.1569780	0.1425840	0.1313060
0.0	**1**	**0.4**	**0.1**	0.2	0.3	0.0877126	0.1043780	0.0946780	0.0870713
0.1	**1**	**0.4**	**0.1**	0.2	0.3	0.0427437	0.0508753	0.0460722	0.0423006
− 0.1	**2**	0.4	0.1	**0.2**	**0.3**	0.1866350	0.2100200	0.1860910	0.1671470
0.0	**2**	0.4	0.1	**0.2**	**0.3**	0.1275180	0.1426770	0.1261100	0.1129770
0.1	**2**	0.4	0.1	**0.2**	**0.3**	0.0639906	0.0711466	0.0627137	0.0560203
− 0.1	1	**0.8**	0.1	0.2	0.3	0.1956250	0.2408200	0.2220010	0.2077540
0.0	1	**0.8**	0.1	0.2	0.3	0.1309950	0.1604810	0.1466300	0.1379340
0.1	1	**0.8**	0.1	0.2	0.3	0.0642991	0.0783394	0.0719247	0.0670413
− 0.1	1	0.4	**0.3**	0.2	0.3	0.3195510	0.3721490	0.3339250	0.3036960
0.0	1	0.4	**0.3**	0.2	0.3	0.2745800	0.3180090	0.2846610	0.2582430
0.1	1	0.4	**0.3**	0.2	0.3	0.2290240	0.2637670	0.2355300	0.2131240
− 0.1	2	0.4	0.1	**0.6**	0.3	0.3190200	0.3562930	0.3124240	0.2768620
0.0	2	0.4	0.1	**0.6**	0.3	0.2148990	0.2392250	0.2094960	0.1853950
0.1	2	0.4	0.1	**0.6**	0.3	0.1063670	0.1179690	0.1031530	0.0911401
− 0.1	2	0.4	0.1	0.2	**0.5**	0.1866350	0.2100200	0.1860910	0.1671470
0.0	2	0.4	0.1	0.2	**0.5**	0.1275180	0.1426770	0.1261100	0.1129770
0.1	2	0.4	0.1	0.2	**0.5**	0.0639906	0.0711466	0.0627137	0.0560203

## Conclusions

Here, we addressed the irreversibility and Joule heating features in MHD hybrid nanofluid flow in non-uniform channel under the influence of peristalsis. The aspects of thermal jump and nanoparticles shape are also included. The major founds of this work are outlined below:

- Hartmann number and non-uniformity parameter show opposite behavior of pressure rise.
- The temperature curve grows with respect to the Brinkman number and thermal slip parameter but declines with respect to the nanoparticle shape effects.
- As anticipated, rate of heat transfer rate displays diverse behavior for different nanoparticles shapes.
- The entropy generation intensifies with an increase in Brinkman number, unlike it behavior with temperature ratio parameter.
- Larger non-uniformity parameter intensifies the axial flow velocity in the central region while decrement is noted in velocity near the walls.

## Abbreviations

$$a_{1}$$: Channel width (m)
$$\overline{u},\overline{v}$$: Velocity components (m s^−^^1^)
$$\overline{p}$$: Pressure (kg m^−^^1^ s^−^^2^)
$b$: Peristaltic wave amplitude (m)
$$\lambda$$: Wave length (m)
$$\overline{T}$$: Fluid temperature (K)
$B_{0}$: Applied magnetic field (A m^−^^1^)
$$Br$$: ^*^Brinkman number
$\beta$: ^*^Heat absorption coefficient
$\gamma$: ^*^Thermal slip parameter
$$Be$$: ^*^Bejan number
$\rho_{hnf}$: Effective density (kg m^−^^3^)
$Q_{0}$: Heat absorption coefficient (W m^−^^2^ K^−^^1^)
$\sigma_{hnf}$: Effective electric conductivity (kg^−^^1^ m^−^^3^ s^−^^3^ A^2^)
$\mu_{hnf}$: Effectivc dynamic viscosity (kg m^−^^1^ s^−^^1^)
$\kappa_{hnf}$: Effectivc thermal conductivity (kg m s^−^^3^ K^−^^1^)
$$\psi$$: Stream function (m^2^ s^−^^1^)
$$T_{0}$$: Reference temperature (K)
$\left( {c_{p} } \right)_{hnf}$: Effective heat capacity (m^2^ s^−^^2^ K^−^^1^)
$$M$$: ^*^Hartmann number
$$\theta$$: ^*****^Dimensionless temperature
$${\text{Re}}$$: ^*^Reynolds number
*: Represents dimension less parameters
